# Screening musicality in children: Development and initial validation of a new tool for rapid assessment of musical profiles

**DOI:** 10.1371/journal.pone.0317962

**Published:** 2025-03-05

**Authors:** Verena Buren, Daniel Müllensiefen, Franziska Degé

**Affiliations:** 1 Department of Music, Max Planck Institute for Empirical Aesthetics, Frankfurt/M, Germany; 2 Department of Psychology, Goldsmiths, University of London, London, United Kingdom; Duervation, AUSTRIA

## Abstract

Musical development in childhood follows universal trajectories, such as the acquisition of basic rhythm and pitch recognition, alongside individual differences shaped by environmental, educational, and innate factors. Despite the importance of understanding these aspects for both research and educational purposes, there remains a significant gap in the availability of validated tools that can quickly and comprehensively assess musicality in children. To address this gap, this paper presents a series of studies on the development and validation of the Child Musicality Screening, a standardised instrument for the assessment of musicality in children aged 3 to 10 years. In Study 1, an initial pool of items was compiled and administered to 810 adults (293 English-speaking, 517 German-speaking) who regularly interact with children. Factor analysis was used to reduce the number of items and to identify three key dimensions of child musicality: *Enthusiasm and Motivation*, *Music Perception*, and *Music Production*. In Study 2, confirmatory factor analysis on ratings of parents (*n* =  305) and educators (*n* =  250) indicated moderate to high model fit, confirming the factor structure of the questionnaire. Interrater agreement between parents and educators was significant, with moderate agreement on the total scale and subscales. Preliminary evidence of convergent and divergent validity was also obtained. Study 3 further established the convergent and divergent validity, as well as internal reliability and test-retest reliability, of the instrument, using both English (*n* =  323) and German (*n* =  384) samples. Overall, the Child Musicality Screening is a newly developed tool for assessing individual musical profiles in children aged 3 to 10 years, with initial evidence supporting its validity and reliability. Available in both English and German, it offers a promising approach for researchers and educators to assess musicality, map developmental trajectories, identify musically gifted children, and enhance targeted music education.

## Introduction

Scientific studies of musical development have generated extensive and remarkable knowledge on the emergence and development of musical perception and production abilities (for a comprehensive review, see [1]). These studies mainly focused on identifying general developmental trends, often delineated by age-related milestones. In recent years, however, there has been a growing emphasis on individual differences in musical development, with attention shifting to variations in ability profiles [e.g., 2–4]. It is increasingly recognised that musical abilities vary between children and do not necessarily develop at the same pace, with substantial individual differences [2].

In addition to evolving research priorities, a major challenge in musical development research is the lack of clear and common construct definitions [5], which complicates the generalisation of results across studies. This issue stems from the inherently multifaceted nature of musicality and the resulting variety of methods and measures employed [6]. Recent efforts have responded to this challenge by conceptualising musicality as a social construct, acknowledging its contextual variability across different cultures, subgroups, and individuals [5,7]. The results of these studies suggest that conceptions of musicality in both adulthood [5] and childhood [7,8] encompass a broader range of skills and behaviours than those typically assessed by traditional musicality tests, which often focus on music perception skills, such as the discrimination of simple musical parameters. Indeed, conceptions of children’s musicality held by parents, educators, and other stakeholders invested in music education extend beyond musical abilities in the narrower sense (perception and production skills) to include facets such as enthusiasm and motivation, musical communication, and analytical understanding of music as important indicators of musicality in childhood [7].

However, this broader conception of musicality has not yet led to the development of corresponding measurement instruments for rapid and scalable screening of general musicality in children that are psychometrically robust and construct-validated. While the Gold Musical Sophistication Index (Gold-MSI) provides a comprehensive measure for assessing musicality in adults [9], few comparable instruments exist for evaluating child musicality. For instance, the Child Musical Behaviour Inventory [CMBI; 10] provides a comprehensive yet fairly lengthy measure for assessing musical behaviour in children under the age of 5 years, while the Music@Home questionnaire [M@H; 11] is specifically designed to assess children´s musical home environments. However, research still lacks reliable and valid measures that are both rapid to administer and able to capture the multifaceted nature of musicality across larger parts of childhood [7].

To address this gap, this study aims to develop a standardized brief screening instrument for the assessment of musicality in children aged 3 to 10 years, that is based on a comprehensive understanding of musicality, meets common psychometric criteria, and is suitable for mapping individual developmental trajectories.

### Musical development in childhood

Every human being has an innate potential or talent to develop musical skills and abilities [12]. Hence, all children have the potential to express themselves musically [13]. However, from the very beginning, developmental processes are driven by enculturation and training [14]. From the very beginning, developmental processes are shaped by socialisation, which encompasses both cultural and social aspects. Musical enculturation, as a part of socialisation, refers to the implicit and effortless acquisition of musical competence through everyday exposure to the forms of music that are typical for the culture one is born into [12]. In contrast, musical training involves formal learning through structured education and practice [15]. In infancy and early childhood, enculturation predominates and active training plays a secondary role [16].

From the third trimester of pregnancy, the foetus is able to perceive and process auditory stimuli [17]. After birth, children already possess a surprisingly sophisticated perception ability: infants are competent music listeners. For example, new-borns and young infants can discriminate simple rhythmic patterns [18–20], and two-month-olds can discriminate between melodies of different length [19]. In terms of pitch processing, sensitivity to melodic contour and relative pitch emerges in early infancy [21], while sensitivity to harmony reaches adult-like performance levels in later childhood [e.g., 22]. Recent research indicates that the development of production skills builds on perceptual skills and progresses throughout childhood [23]. While production skills emerge later than perceptual skills, they are already evident from early stages of development. Precursors of music production emerge before a child’s first birthday, including rhythmic movements in response to music and early song-like vocalisations [24,25]. In their musical development infants are universalists, meaning that they are perceptually equipped for music from any culture [26]. Their resolution of pitch [27] and timing [28] allows them to detect the smallest musically meaningful differences from their own culture and others [12,29]. During the first year of life, infants become attuned to the music that surrounds them and become increasingly proficient with patterns from their native culture [29].

By the time children enter school at around the age of 6 years, they demonstrate fundamental universal and basic culture-specific musical competence [21]. Commonplace musical competence is acquired effortlessly [12] through everyday musical interaction such as singing songs, dancing, playing musical games, and exposure to recorded music [11], for a review, see [30]. These musical interactions are frequent and highly complex [31], and the quantity and quality of informal musical activities may have specific influence on children’s social, cognitive and language skills [11].

Children first develop basic music competencies, such as the sensitivity to melodic contour and relative pitch [21], as well as the ability to discriminate simple rhythmic patterns [18–20]. During primary school age, they achieve greater perceptual sophistication and complexity [32], including recognising more subtle pitch and sound characteristics [e.g., [32,33]. Additionally, tempo discrimination [34] and the ability to adapt to changes in meter [35] improve significantly. Production skills also advance during this period, as evidenced by greater accuracy in keeping a steady beat [36] and synchronized tapping [34]. Enculturation is complete at around the age of 10 to 12 years of age [16], when many music-related competencies are so well developed that they are similar to those of untrained adults [37]. Beyond enculturation, formal music training in the Global North often starts during kindergarten or primary school age [16]. These musical experiences, especially through instrument learning, can enlarge individual differences in the in the course of development [37]. In summary, some musical competencies are present from the very beginning of life, while others reach a higher level of knowledge and specialization only in older and musically experienced individuals [32].

While children universally acquire musical competencies and the basic principles of their development are increasingly understood, conclusions have predominantly been drawn from sample averages and aggregated values rather than individual data [38]. The traditional approach in developmental music psychology has largely centred on identifying common patterns, such as average improvements through music interventions or age-related average musical abilities in a given sample. Although this approach has been invaluable in advancing our understanding of general developmental trends, it may overlook the remarkable variability in individual developmental trajectories and the diverse manifestation of musical abilities.

Recent research on individual differences in musical ability has highlighted the roles of genetic factors and gene-environment interactions. It has been shown that even in individuals without formal musical training, musical ability (measured by a perception test) can reach musician-like levels and is associated with informal musical experience and performance on cognitive tests [39]. Furthermore, music perception ability was found to predict other variables such as recognising vocal emotions, regardless of musical training [40]. A musically enriched childhood environment was also found to amplify individual differences, likely by providing opportunities that allow genetic factors to play a more prominent role in shaping outcomes [4]. These results challenge the view that learning and practice are the central origin of musical ability [41] by documenting genetic contributions to numerous aspects of musical abilities and behaviours in the general population.

In children, early musical ability was found to be associated with the duration of music training over the subsequent five years and to influence the likelihood of pursuing, adhering to, and benefiting from musical training [2]. Despite significant improvements in musical ability during this period, the stability in individual performance (in terms of who performed well or poorly) suggested that the impact of music training might be less significant than previously assumed [2]. Conversely, a study involving a large sample of children aged 9 to 17 years found that the amount of musical training did indeed positively affect the development of musical abilities over time [3].

This line of research underscores the importance of individual data in uncovering gene-environment contributions to musical ability, revealing that phenotypic musical ability results from a genetic profile that includes certain predispositions working in combination with environmental factors. Assessing individual musical profiles across different dimensions of musicality provides valuable insights into a child’s strengths and potential areas for development, helping to better understand their unique musical trajectory. This approach contributes to our understanding of individual developmental pathways and underlying mechanisms, thereby filling a gap in current knowledge about musical development. However, this line of research also requires clear conceptions of musicality and measurement tools that meet stringent psychometric standards.

### Conceptions of musicality

The assessment of musicality has been a research interest since at least the middle of the last century, yet consensus on the core components of musicality and their interrelationships remains elusive. Despite advancements in understanding the development of individual musical skills, the broader scope of developmental trajectories and the interconnectedness of these skills with the concept of musicality remain unclear.

A major challenge has been the lack of a precise definition of musicality or musical ability, which has led to the use of various test procedures based on different models of musicality. For example, the Seashore Measures of Musical Talent [42] are based on a multi-faceted conception of musicality, assuming that the musical profile of an individual consists of several and only loosely related aspects of musicality. Seashore used the term ‘talents’ and provided tests for, e.g., the discrimination of single pitches, short melodies, rhythms, etc. On the contrary, Bentley’s Measures of Musical Abilities [43] assume that musical ability is best described by a general musical ability factor, assuming that all musical facets are substantially interrelated. These differences in conceptualisation can lead to extremely different music tests with some solely focusing on the sub-competencies that are considered fundamental by the test authors [44,45]. This, in turn, contributes to inconsistent psychometric results and low validity in existing measures [e.g., 45–48]. In addition to those conceptual issues, there has also been criticism of the tendency of testing procedures to overly focus on perceptual skills (e.g., same/different judgments of melodies or rhythms) and on aspects that are captured in music notation, while paying much less attention to production tasks, such as singing or tapping along to music. Furthermore, the majority of existing assessment batteries do not consider broader skills and behaviours such as musical communication, understanding, and motivation, that are considered highly critical for later musical development [49], thus limiting their ability to provide a comprehensive approach that acknowledges the multifaceted nature of musical behaviour [6]. While practical considerations may partly explain this imbalance, the absence of a common concept of musicality has led to mixed results, that leave it unclear how different measures or scores relate to each other, limiting the ability to generalise across tasks and results [6].

Moreover, it is important to recognise that existing models and tests are predominantly Eurocentric or Western-oriented. This focus can limit the applicability and relevance of these assessments across different cultural contexts. One approach to overcome conceptual controversies is to consider musicality as a social construct, incorporating the assumption that musicality is not an inherent, universal quality, but rather a concept defined and redefined by society [5]. This implies taking into account the conceptions of musicality held by the general population, as well as by musicians and music educators from a specific period and region, thereby acknowledging the cultural specificity and the variability of such conceptions over time. In a series of studies, Hallam and colleagues have explored the conceptions of musicality held by people from different musical backgrounds [5,47,50,51]. In an initial qualitative study, Hallam and Prince [5] identified six overarching themes that adults and students associate with musicality, namely: aural skills, receptive activities, generative activities, the integration of a range of skills, personal qualities, and the debate about innate versus learned musical ability. Subsequently, Hallam used 77 statements generated from the qualitative study for a quantitative study of musical conceptions [51]. PCA analysis revealed six components of musicality: playing instruments or singing, musical communication, valuing and responding to music, composition and improvisation, commitment and motivation, and rhythmic ability and pitch understanding.

To explore whether these conceptions of musicality extend to childhood and infancy, Buren et al. [7] adapted the statements used by Hallam [51] to align with the abilities of 3- to 6-year-olds and 922 German adults rated the resulting 49 statements according to the frequency they believed a musical child would exhibit these behaviours. PCA analysis identified four components of children’s musical abilities: Musical Communication, Enthusiasm and Motivation, Analytical Understanding, and Musical Abilities (in a narrower sense, i.e., perception and production skills). Only participants’ ratings of analytical understanding were influenced by their musical and pedagogical training. For infancy and toddlerhood, three of the four components were replicated (Musical Communication, Enthusiasm and Motivation, and Musical Abilities) [8]. The component Analytical Understanding of Music was replaced by Adaptive Expressiveness for the younger ages, reflecting a less cognitive and more intuitive understanding of music. Although these components thus differ in their focus, they fundamentally measure a similar underlying concept. For younger children, Adaptive Expressiveness captures the spontaneous and instinctive ways they engage with music, whereas Analytical Understanding is more suited to older children who can cognitively process musical structures and concepts. In both studies, enthusiasm and motivation were consistently rated as the most significant indicators of musicality in childhood [7,8].

Taken together, these findings suggest that musicality as a social construct is multifaceted and that conceptions differ between adult and child musicality. While both conceptions of musicality emphasise the significance of musical communication and motivation besides musical abilities in a narrower sense, differences emerge in the complexity of the conceptions: while Hallam’s research identified six components of musicality in adults [51], children’s musicality appears to be constituted by only four distinct components [7]. Thus, when measuring musicality in childhood, it is important to recognise that qualitatively different abilities may play a role, and that the measurement should not be based solely on criteria used to assess musicality in adults. In addition, in order to accommodate a broader definition of musicality into the assessment of musicality, it is important to consider a wide range of skills from an early age.

### Questionnaires and screening tools on musicality in childhood

Incorporating the multifaceted nature of musicality into a standardised test or battery of tests presents a significant challenge, but questionnaires and screening tools offer a convenient and time-efficient means of capturing more complex behaviours. However, attempts in this direction are surprisingly scarce. The most prominent and widely adopted example of a questionnaire that is based on a broader understanding of musicality is the Goldsmiths Musical Sophistication Index (Gold-MSI), a self-report inventory for adults that assesses individual differences in musical sophistication in non-musicians [52]. For children, there is a slightly adapted version that has been validated for secondary school age [53]. However, self-reporting becomes increasingly difficult in earlier childhood. Therefore, it is common to rely on parent or educator assessments of child behaviour. Although this approach seems relatively straightforward, there have been limited attempts to develop measurement tools for assessing musical abilities and behaviours.

Currently, there are two parent-report questionnaires that explore musical practices in the home environment: The Home Musical Environment Scale [HOMES; 54], a fifteen-item questionnaire designed for parents of school-aged children and the Music@Home questionnaire [M@H; 11], available for parents of preschool children (2–5.5 years; 17 items) and infants (3–23 months; 18 items).

Focusing on educational implications, the Musical Development Matters Inventory [55] is a checklist designed to provide educational guidance by documenting various musical learning moments in early childhood development (from birth to 5 years). As the emphasis of the inventory is on providing interpersonal and environmentally relevant opportunities to promote musical growth, standard practices and principles of test construction have not been applied in its construction. Similarly, the Observation and Assessment Sheet for Competencies and Interests of children (KOMPIK, available only in German) serves as an observation tool in educational contexts. It provides insights into various developmental areas for children aged 3.5 to 6 years, supporting educational action [56]. For musical development, the questionnaire covers a range of behaviours divided into musical interests (7 items) and competencies (8 items). The questions were formulated in close consultation with educators and experts, and subsequent analyses confirmed the two-factor structure of the musicality questionnaire [57].

An example of a musicality screening aimed at identifying highly gifted students is the checklist that is part of the Munich High Ability Test Battery for Secondary Education (MHBT-S) which is available in German [58]. The musicality checklist includes 11 abilities/feelings that are rated for their presence by a teacher, but no psychometric examination has been conducted [58].

Other measures are ad-hoc questionnaires developed as part of research projects to include a measure of musicality in a research study. These measures have typically not undergone rigorous testing to ensure their reliability and validity and aim to collect specific data on aspects such as musical activities, training, or daily musical interactions [e.g., 59–61]. Consequently, the primary aim is not to formulate a comprehensive measure of musicality, and psychometric standards are often not the primary concern.

A validated tool that assesses music-related behaviours in children under the age of 5 years which is available in English is the Children’s Music-Related Behaviour Questionnaire or Child Musical Behaviour Inventory [CMBI; 10]. This parental assessment questionnaire consists of 97 items that assess the frequency of various musical activities such as singing, dancing, and listening to music. There are seven subscales relating to child-initiated musical behaviour (Attention and Emotion, Vocalisations, Moving, Daily Routines, Requests, Taking Turns, Creativity) and one subscale relating to parent-initiated activities. The subscales showed good reliability (Cronbach´s alpha .77–.97) and structural consistency was confirmed by confirmatory factor analysis [10]. However, with its 97 items, the CMBI is very time-consuming and, so far, it has only been published in English. To give researchers as well as practitioners the possibility to assess musicality both comprehensively and efficiently, there is a need for a screening tool based on empirical evidence and aligned with stakeholder views regarding the aspects that a broad conception of children’s musicality should encompass.

### Objective

The primary objective of the present study was to develop a screening tool for assessing individual profiles of general musicality in children aged 3 to 10 years, using questionnaire-based assessments from parents and educators. In the development process, our aim was to keep the screening concise yet grounded in a comprehensive understanding of musicality, while also adhering to rigorous questionnaire development procedures. Our overarching goal is to provide researchers with a tool that facilitates the rapid assessment of musicality, supports the investigation of developmental trajectories, and offers insights into individual variations in musical abilities. While the tool is not designed to provide exhaustive measurement, it can contribute to exploring correlations with other cognitive abilities or environmental influences. In educational settings, the screening can be used by educators to tailor their approaches and ensure that each child can benefit optimally from music education. It can also be used to identify particularly gifted children who may benefit from additional opportunities to further their musical development.

We chose the age group of 3 to 10 years as target age range for the screening for several reasons. Firstly, children under 3 years of age have distinct cognitive, motor, and emotional abilities compared to older children [62–64]. Additionally, research suggests that conceptions of musicality in infants and toddlers differ from those of older children [8]. Secondly, by around the age of 10 to 11, middle to late childhood ends [65], and in many countries children enter secondary school, reading speed has developed considerably, and hence self-assessment becomes more feasible. Therefore, focusing on children between the ages of 3 and 10 years allows us to capture crucial developmental stages while ensuring that the questionnaire remains relevant to a wide age range. The questionnaire has been developed in both English and German to facilitate its use in different countries. It has also been designed to be used by researchers as well as educators, parents, and caregivers, allowing its successful application in a variety of scientific and educational settings.

## Study 1: Development of the Child Musicality Screening (CHIMUS)

In the first study, an initial screening questionnaire was created, consisting of a large number of items that were intended to reflect child musicality comprehensively. The goal was to reduce the items according to psychometric criteria and to create a quick-to-administer screening of child musicality in German and English, that could be used by teachers and parents to assess the musicality of 3- to 10-year-old children. For readability, we will use the term “parents” throughout the text to refer to both parents and primary caregivers.

### Methods

#### Participants.

The study received ethical approval by the Ethics Council of the Max Planck Society (No. 2017_12), and was undertaken with informed consent of each participant. Participants were recruited via the homepage and social media of the Max Planck Institute for Empirical Aesthetics as well as by directly contacting schools and kindergartens. In addition, Prolific (www.prolific.com), an online platform where registered volunteers receive a small compensation for their participation in questionnaire studies, was used for recruitment [66]. To be eligible for the study, the person completing the survey had to spend regular time (at least 10 hours per week) with at least one child between 3 and 10 years of age. A total of 1,121 people participated in the survey (*n* =  649 German-speaking and *n* =  472 English-speaking), however 311 were excluded due to having completed less than half of the survey (*n* =  278), reporting filling out the survey for a child outside the age range (*n* =  31), or giving implausible answers concerning the number of children they regularly spend time with (*n* =  2). The final sample consisted of 810 (*n* =  517 German-speaking, *n* =  293 English-speaking) participants (*n* =  402 female, *n* =  390 male, *n* =  6 diverse, *n* =  12 n/a) between 18 and 81 years with a mean age of 32.93 years (*SD* =  10.43). Eight participants did not indicate their age. Six hundred and twenty-five participants knew the child from a private setting, 185 from a professional setting. The ages of the children for whom the survey was completed ranged from 3 to 10 years, with a mean age of 6.57 years (*SD* =  2.22).

For further analysis, we established two age groups: the first comprising children aged 3-6 years (*n* =  396), and the second consisting of children aged 7–10 years (*n* =  414). These age groups were chosen for compatibility with our previous paper [7] and because children in Germany usually complete the transition to secondary school at the age of 11. In addition, we considered this to be an age at which self-report is easy to conduct and may be more informative than parental report.

#### Materials.

The initial item pool was created in English and drew from multiple sources. It was primarily based on the items developed by Buren and colleagues [7], who had already established a broad definition of musicality. Additionally, items were extracted from various questionnaires on children’s musicality identified through an extensive literature review. Moreover, free-text responses to a query about additional aspects of children’s musicality from Buren et al. (7) were incorporated to develop further items [7]. This comprehensive approach resulted in a list of 242 items that collectively encompassed a wide definition of child musicality.

Item reduction was done in several steps. The decision on which items could be removed was made by consensus between three music psychologists (the authors) and two graduate students working on the project. First, clearly redundant items were removed. The remaining items were first aligned so that they all began with the prefix “The child...”. Further reduction was done by checking comprehensibility and relevance and by removing items that were similar in content, while maintaining the broadest possible content coverage. The final set of 57 items was sourced as follows: 38 items from Buren and colleagues along with 5 additional items from their questionnaire’s open responses [7], 5 items from Burke [55], 6 items from Mayr and colleagues [67], and 3 items from Heller and Perleth [58,68]. To avoid the acquiescence response bias, inverted versions of the items were created and their wording revised. Those 22 items that were easy to understand despite negative wording were then included in the questionnaire in their inverted form. The length of 57 items was considered appropriate for initial empirical data collection (see S1 File for details).

In line with Buren and colleagues [7], a 5-point Likert scale was employed. Depending on the nature of the items, either a frequency scale (ranging from “rarely/never” to “always”) or an agreement scale (ranging from “totally disagree” to “totally agree”) was used. Item scores for negatively worded items were inversely coded. The questionnaire was then translated into German through a parallel translation process conducted by two native German speakers. This was followed by back-translation and resolution of discrepancies through consensus among the authors. In addition to the musicality questionnaire, we collected socio-demographic information about the participant and the child, as well as details regarding the formal relationship between the respondent and the child.

#### Procedure.

Data collection for both the German and English versions was conducted online between May and June 2022 using LimeSurvey [69]. Informed consent was obtained by all participants. A pre-screening ensured that participants were fluent in the language of the questionnaire and regularly interacted with at least one child aged 3 to 10 years. Participants were instructed to think of a single child they knew best and to complete the questionnaire with this child in mind. Then, they responded to the items of the musicality questionnaire along with a demographic questionnaire. In total, the participation took approximately 10-15 minutes. Participants recruited via Prolific received compensation for completing the questionnaire, following a plausibility check.

#### Statistical analyses.

Statistical analyses were performed using RStudio, version 4.4.1 [70] and the packages GPA rotation [71], lavaan [72], psych [73], and semTools [74]. To select the items for the final screening questionnaire, an iterative method was used in which the number of factors to be extracted was determined based on a parallel analysis, consistent with established practices [75–77]. Factors were then extracted using exploratory factor analysis with minimum residuals, guided by iterative parallel analyses that allowed for correlated factors and employing oblimin factor rotation. Items were screened and removed in each iteration based on ambiguous or low factor loadings and low communalities. This analysis was based on the German-speaking sample only. As a final step, we tested for invariance between the German and English questionnaire, and between the two age groups (3 to 6 years and 7 to 10 years).

#### Results of study 1.

For the initial rounds of iterations, we conducted an exploratory factor analysis, with the number of factors determined by parallel analysis with 200 simulated datasets. From the results of this analysis, 32 items that loaded on at least one factor with ≥ .3 and had a communality of ≥ .4 were kept for the next iteration with the same criteria. After the second iteration, there were no further changes. Since 32 items was deemed too long for a short questionnaire, a stricter criterion of factor loading of value ≥ .4 on at least one factor was applied for another iteration, while keeping the required communality still at ≥ .4. After conducting three more parallel analyses and EFAs, no further changes were observed in the data, while the number of items was still at 28. Finally, to enhance the interpretability of the factors, an additional criterion was introduced, whereby items should load maximally at one factor. After six more iterations, a preliminary solution with 19 items on three factors was found. Both the relative (TLI, CFI) and absolute fit indices (RMSEA, SRMR) of this model were in an acceptable to good range (RMSEA = .05, CFI = .96, TLI = .95, SRMR = .04).

To determine a questionnaire that exhibits similar characteristics for both language groups surveyed (German and English), a confirmatory factor analysis (CFA) with invariance tests was conducted for the two subsamples of each language. The fit indices CFI and RMSEA at the level of configural invariance, as well as *p-*values of *χ*² tests, ΔRMSEA, and ΔCFI, were used as indicators for assessing invariance. The latter three values describe the relationships between values at different levels of invariance with increasingly stringent assumptions [78–80]. The following assumptions regarding the groups for the corresponding levels of invariance apply: (1) for configural invariance, the equality of the factor structure; (2) for metric or weak invariance, the equality of factor loadings; (3) for scalar or strong invariance, the equality of factor loadings and intercepts. Other types of invariance testing (e.g., strict invariance or invariance of latent means) were not deemed necessary or helpful to assess in the context of a confirmatory factor analysis following Little [81]. A widely used criterion for empirical invariance testing is a ΔCFI value of no greater than .01 between subsequent levels of invariance [82]. This benchmark was exceeded for the initial 19-item model across languages (German and English) at the level of scalar invariance (see Table 1), hence, scalar invariance could not be established.

**Table 1:** Test of invariance for the initial model across languages.

Level of invariance	CFI	RMSEA	ΔCFI	ΔRMSEA	χ²	p(χ²)
**Configural invariance**	.95	.06			677.55	
**Metric invariance**	.95	.06	.00	.00	704.64	.041
**Scalar invariance**	.93	.07	.02	.01	909.03	<.001

CFI =  Comparative Fit Index, RMSEA =  Root Mean Square Error of Approximation, ΔCFI =  difference in CFI between the current stage of invariance and the previous stage of invariance, ΔRMSEA =  difference in RMSEA between the current stage of invariance and the previous stage of invariance.

Therefore, we modified the model by removing further items using several strategies. Factor loadings and item variance homogeneity, assessed by Levene’s tests with language as the grouping variable, guided these modifications. Several alternative models (A) were checked: (1) Model A1 involved selecting the four items per factor with the highest factor loadings, (2) Model A2 involved selecting the three items per factor with the highest factor loadings, (3) Model A3 involved selecting items with factor loadings greater than or equal to .7, (4) Model A4 involved selecting items with homogenous variance (Levene’s test *p*-values greater than .05), and (5) Model A5 involved selecting the three items per factor with the highest *p*-values, provided they were at least .05. (6) For model A6, only homoscedastic items (according to Levene’s test) with a factor loading of at least .5 were selected. This approach aimed to optimize both the robustness of the factor structure and the interpretability of the results. To determine the final model, substantive considerations were integrated to ensure that the derived factors were both interpretable and aligned with the predefined criteria. Model A6 with 9 items was selected as the final model, as it fulfilled these criteria effectively. The factor loadings of the final model are displayed in Table 2, while the results of the invariance tests across languages for the final model are presented in Table 3.

**Table 2:** Factor loadings of the final model.

	Factor loading		
Item	Factor 1	Factor 2	Factor 3
Das Kind…/The child…			
…hat oft den Wunsch, Musik zu machen.^1^ …often has the desire to make music.^1^	**.82**	.01	−.05
…hat große Begeisterung für Musik.^2^ …has great enthusiasm for music.^2^	**.76**	.03	.04
…genießt Musikmachen als Teil seines Lebens.^2^ …enjoys making music a part of his/her life.^2^	**.79**	.01	.04
…hat ein gutes Gespür für Timing und Rhythmus.^1^ …has a good sense of timing and rhythm.^1^	.03	**.86**	−.01
…hat ein Gefühl für den Takt.^1^ …has a feeling for the beat.^1^	−.04	**.80**	.01
…hat eine gute Hörfähigkeit, z.B. für Melodien und Rhythmen.^1^ …has good hearing ability, e.g., for melodies and rhythms.^1^	.17	**.55**	.10
...hat Schwierigkeiten, Musik zu produzieren oder zu reproduzieren.^2^ ...shows difficulties in producing or reproducing music.^2^	.09	−.07	**.82**
...passt beim Musikmachen wenig auf, sodass es nicht merkt, ob es so klingt wie beabsichtigt.^2^ ...pays little attention while making music, so he/she does not realize if it sounds as intended.^2^	.03	.10	**.54**
…hat Probleme, Melodien, die es zuvor gehört hat, zu reproduzieren.^2^ …has issues reproducing melodies he/she has heard before.^2^	−.11	.12	**.71**

^1^agreement scale,

^2^frequency scale.

**Table 3:** Test of invariance for the final model across languages.

Level of invariance	CFI	RMSEA	ΔCFI	ΔRMSEA	χ²	p(χ²)
**Configural invariance**	.98	.05			102.32	
**Metric invariance**	.98	.05	.00	.00	106.22	.691
**Scalar invariance**	.97	.06	.01	.01	136.78	<.001

CFI =  Comparative Fit Index, RMSEA =  Root Mean Square Error of Approximation, ΔCFI =  difference in CFI between the current stage of invariance and the previous stage of invariance, ΔRMSEA =  difference in RMSEA between the current stage of invariance and the previous stage of invariance.

In addition to the different language groups considered in the model selection, the sample also comprised various age groups, including children at different developmental stages. To ensure the model’s applicability across different age ranges, it was tested for invariance across the predefined age groups of 3 to 6 years and 7 to 10 years. The final model satisfied the criteria for invariance across these age groups as well (see Table 4).

**Table 4:** Test of invariance for the final model across age groups (3 to 6 years, 7 to 10 years).

Level of invariance	CFI	RMSEA	ΔCFI	ΔRMSEA	χ²	p(χ²)
**Configural invariance**	.99	.04			77.98	
**Metric invariance**	.99	.03	.00	.01	79.11	.980
**Scalar invariance**	.99	.04	.00	.01	96.74	.007

CFI =  Comparative Fit Index, RMSEA =  Root Mean Square Error of Approximation, ΔCFI =  difference in CFI between the current stage of invariance and the previous stage of invariance, ΔRMSEA =  difference in RMSEA between the current stage of invariance and the previous stage of invariance.

The final version of the questionnaire was named Child Musicality Screening (CHIMUS) and reveals three distinct factors that capture various dimensions of musicality. The first factor, termed Enthusiasm and Motivation, reflects a child’s intrinsic motivation and emotional response to music, highlighting their general excitement and enjoyment in making and integrating music into their daily life. The second factor, Music Perception, assesses a child’s perceptual skills, including their sense of rhythm, timing, and auditory discrimination. The third factor, Music Production, identifies difficulties related to music-making and reproduction. It encompasses challenges such as attention issues during music-making, problems with reproducing melodies, and overall difficulties in music production. The final model demonstrated good fit indices (RMSEA = .05, CFI = .98, TLI = .98, SRMR = .03). The factors were moderately to highly correlated with each other (*r*^factor1, factor2^ = .56, *r*^factor1, factor3^ = .52, and *r*^factor2, factor3^ = .66).

While the factor model does not include a general factor, the strong correlations between the subscales indicate that a total score may be used to calculate an overall assessment of a child’s musicality. According to Sijtsma and colleagues [83], it is not necessary for all items to load onto a single latent factor to justify the use of a total score. In fact, a sum score can serve as a reliable and valid measure even when multiple dimensions are present. The Child Musicality Screening thus allows for the use of both subscale scores and an aggregated total score. The total scale exhibited good to very good internal consistency as indicated by Cronbach’s α (α = .86, 95% CI [0.85, 0.88]) and by the McDonald’s ω_total_ (ω = .90; 95% CI [0.90, 0.96]). The α and ω of the subscales Enthusiasm and Motivation and Music Perception were also high, while the Music Production subscale demonstrated acceptable reliability (see Table 5).

**Table 5:** Reliability coefficients of the subscales of the final model.

	CHIMUS Total Scale	Factor 1 Enthusiasm & Motivation	Factor 2 Music Perception	Factor 3 Music Production
**α**	.86	.82	.82	.73
**ω**	.90	.83	.83	.75

**α** = Cronbach´s α [84], **ω** = McDonald’s ω_total_ [85].

## Study 2: Assessing the adequacy and interrater reliability of the Child Musicality Screening

In the second study, the new Child Musicality Screening (CHIMUS) was validated using an independent sample. In addition to replicating the factor structure with a new dataset, the goal was to establish interrater reliability by comparing teacher and parent ratings of the same child. The third goal was to gain initial evidence for convergent and divergent validity.

### Methods

#### Participants.

The study received ethical approval by the Ethics Council of the Max Planck Society (No. 2017_12), and was undertaken with informed consent of each participant. Schools and kindergartens in Germany were recruited through email and personal contacts. Once a school or kindergarten had agreed to participate, the parents of children aged 3 to 10 were contacted through these institutions. Although the goal was to obtain assessments from both a parent and a teacher for each child, ratings for a child from only a single source were also included (in order to obtain a sufficiently large sample for the assessment of the factor model). A total of 308 parents participated in the survey (244 female, 56 male, 8 n/a), aged 24 to 59, with a mean age of 39.73 years (*SD* =  5.98), 9 parents did not indicate their age. Correspondingly, 261 assessments were provided by teachers. Of these, 154 assessments were attributed to 13 teachers (each completing between 1 and 16 assessments except one, who completed 31), while the remaining 96 teacher ratings could not be attributed to specific teachers due to missing codes. Similarly, analysis of their demographic data was not possible as the majority of teachers did not provide the required information. All participating institutions were located in Hesse or Hamburg, Germany. The mean age of the children assessed was 6.23 years (*SD* =  2.19). Three parent ratings and 4 teacher ratings were removed from the analysis because less than 50% of the Child Musicality Screening had been completed. Additionally, data from one teacher, comprising 7 assessments, were excluded due to negative correlations with the parent assessments, suggesting a potential misuse of the scale. Thus, the final dataset included 305 parent assessments and 250 teacher assessments, with 247 corresponding ratings.

#### Materials.

The questionnaire was administered in paper format. Two versions of the German version of the CHIMUS were created: one for educational staff, prefixed with “the child”, and another for parents, prefixed with “my child”. Following the structure of the Child Musicality Screening, a 5-point Likert scale was employed, either as a frequency scale (ranging from “Rarely/Never” to “Always”) or as an agreement scale (ranging from “Strongly disagree” to “Strongly agree”). Negatively worded items were reverse-coded. Additionally, socio-demographic data were collected, along with information regarding the child’s most recent school grade in music (school children only). The German school grades were inversely coded, so that higher scores indicate better performance.

In 117 cases, the questionnaire was administered as part of a larger set of questionnaires, because these children took part in another study. Consequently, their parents also completed the German version of the Music@Home questionnaire [86], the German version of the CBQ in its very short form [87], and an adapted version of the BFI-10 [88]. We used the German version of the Music@Home Preschool questionnaire [86] which comprises 17 items encompassing Parental Beliefs, Child Engagement with Music, Breadth of Musical Exposure and Parent Initiation of Musical Behaviour. The very short form of the CBQ [87] comprises 36 items on the scales Negative Affectivity, Surgency Extraversion, and Effortful Control. The BFI-10 is a brief measure of the Big Five personality traits, consisting of ten items that assess the dimensions Neuroticism, Extraversion, Openness to Experience, Agreeableness and Conscientiousness. It was adapted by the authors of this manuscript in order to be used as a parent questionnaire reporting on the personality of their children.

#### Procedure.

Data collection took place between January and November 2023. Paper questionnaires were distributed to participating schools and kindergartens, which then forwarded them to parents along with an information letter detailing the study and data protection measures. Parents who consented to participate signed the consent form and completed the questionnaires. The educational staff collected the completed forms. For children whose parents had agreed to participate, teachers also signed a consent form and filled out a teacher questionnaire, along with a form about their own demographic data and musical background. The schools or kindergartens then returned all forms to the researchers. Participation in the study took approximately 10–20 minutes. No compensation was provided to participants.

#### Statistical analyses.

Statistical analyses were performed using RStudio, version 4.4.1 [70]. Packages used were lavaan [72], psych [73], and diffcor [89]. First, inverted items were recoded. In a next step, the total score of the CHIMUS was calculated as sum score of all items. In addition, subscale totals were calculated by adding up the values from the respective three items of the subscale. These were only formed if none of these values were missing.

Confirmatory factor analysis (CFA) was applied to assess the factorial validity of the newly developed questionnaire using the new sample. To determine whether the factor structure was consistent between parent and teacher data, analyses were performed separately for both groups. Missing data were handled using Full Information Maximum Likelihood (FIML), allowing the use of all available data without the need for imputation. Internal reliability of the parent and teacher ratings was assessed using Cronbach’s alpha and McDonald’s omega total. Interrater reliability was evaluated through Pearson correlations.

To gain initial evidence of convergent validity, we performed Pearson correlations between the Child Musicality Screening and school grades as well as the Music@Home questionnaire. For divergent validity, we assessed the CHIMUS’s correlation with the Extraversion scale (2 items) from the BFI-10 and the Surgency scale (12 items) from the CBQ. We expected no significant correlations with BFI-10 Extraversion nor with CBQ Surgency, as musicality should not be solely attributable to personality traits related to impulsivity, activity level, or pleasure intensity.

#### Results of study 2.

In order to assess the adequacy of the factor model, a CFA was conducted. The fit indices for parents and educators are presented in Table 6. The analysis revealed that the model fit was slightly better for educators compared to parents. Specifically, the educator data demonstrated fit indices that were mostly in the good range, while the indices for parents were generally in the acceptable range. This suggests that the model fits the educator data slightly better than the parent data, according to both relative and absolute fit indices.

**Table 6:** Fit indices of the rater groups (parents, educators).

	Parents	Educators	Δ_Parents−Educators_
**χ²**	82.71	66.16	
**df**	24	24	
**RMSEA**	.089	.083	0.006
**CFI**	.949	.973	−0.024
**TLI**	.924	.959	−0.036
**SRMR**	.061	.038	0.023

CFI =  Comparative Fit Index, RMSEA =  Root Mean Square Error of Approximation, SRMR =  Standardized Root Mean Square Residual, TLI =  Tucker-Lewis Index

Following the CFA, internal consistency was assessed for both groups (see Table 7). The results indicate that the internal consistency of the CHIMUS is high across both educator and parent ratings, with particularly strong reliability observed in the educator group. The Production subscale showed slightly lower, yet still acceptable, reliability.

**Table 7:** Internal consistency of educator and parent ratings.

	α_educators_	α_parents_	ω_educators_	ω_parents_
**Total Scale**	.95	.86	.95	.91
**Motivation Subscale**	.90	.81	.90	.82
**Perception Subscale**	.90	.84	.90	.87
**Production Subscale**	.82	.78	.83	.78

α = Cronbach’s Alpha [84], ω = Omega total [85]

Interrater correlations of educator and parents were assessed. It reached *r* = .46, *p* < .001, *n* =  207, for the overall scale and *r*_motivation_ = .43, *p* < .001, *n* =  231, *r*_perception_ = .36, *p* < .001, *n* =  239, and *r*_production_ = .34, *p* < .001, *n* =  219 for the subscales. These findings suggest a moderate, but highly significant agreement between educators’ and parents’ assessments, particularly evident in the overall scale scores.

In addition, we examined the correlation between the CHIMUS and the most recent school grade in music, as reported by the parents. Pearson correlations indicated moderate but significant relationships, with the total scale showing *r* = .46, *p* < .001, *n* =  83, and subscale correlations ranging from .30 to .40 (see Table 8). Further support for convergent validity was obtained by correlating the CHIMUS with the Music@Home scale, which was also completed by the parents. The total score of the CHIMUS showed a strong correlation with the Music@Home General factor (*r* = .52, *p* < .001, *n* =  96), with the highest correlation observed for the Enthusiasm and Motivation subscale (see Table 8).

**Table 8:** Convergent validity of the Child Musicality Screening with school grades and the Music@Home General factor, and divergent validity with extraversion (BFI-10) and surgency (CBQ).

	School grade	M@H General factor	Extraversion (BFI-10)	Surgency (CBQ)
CHIMUS	.46*** (83)	.52*** (96)	.05 (101)	.00 (61)
Enthusiasm & Motivation	.30** (89)	.55*** (101)	.10 (107)	.07 (64)
Perception	.36*** (87)	.34*** (103)	.01 (110)	.04 (64)
Production	.40*** (84)	.41*** (97)	.02 (103)	−.09 (61)

Pearson correlations (two-tailed). Values in parentheses indicate sample size (*N*). CHIMUS =  Child Musicality Screening, M@H =  Music@Home, BFI-10 =  Big Five Inventory 10, CBQ =  Child Behaviour Questionnaire. *  =  *p* < .05; ** =  *p* < .01; *** =  *p* < .001. Sample sizes (*N*) vary due to missing data in some variables.

To assess divergent validity, we evaluated the correlation of the CHIMUS with the Extraversion scale of the BFI-10 and the Surgency scale of the CBQ (very short version). The parent ratings of their child´s musicality did not significantly correlate with their assessment of their child’s extraversion (*r* = .05, *p* =  601, *n* =  101) or the reported surgency of the child (*r* = .00, *p* = .980, *n* =  61). Additionally, there was no significant relationship between Extraversion/Surgency and the Enthusiasm and Motivation subscale, indicating that enthusiasm for music is not primarily driven by general extraversion, or related qualities like impulsivity, activity level, or pleasure intensity.

## Study 3: Validity and test-retest reliability of the Child Musicality Screening

Study 1 and 2 developed and confirmed the adequacy of the item and factor structure of the Child Musicality Screening and examined the interrater reliability of parents and teacher ratings. Initial evidence also pointed towards the validity of the measure. As the factor model had previously only been confirmed for the German version, our aim in Study 3 was to confirm the factor model for both language versions. In addition, we aimed to analyse the convergent validity with further measures as well as the retest reliability.

### Methods

#### Participants.

The study received ethical approval by the Ethics Council of the Max Planck Society (No. 2017_12), and was undertaken with informed consent of each participant. Participants for the online study were recruited through the website and database of the Max Planck Institute for Empirical Aesthetics, as well as via personal contacts and Prolific (www.prolific.com). To expand the educator group, 68 German-speaking trainee educators from a dual-track practice-integrated program, alternating between school instruction and childcare workdays, completed a paper version of the questionnaire. Unlike in Study 2, no overlap between educators and parents was planned, as interrater reliability was not a focus of this study. To qualify for the study, individuals needed to spend regular time with a child aged between 3 and 10 years, and be fluent in either German or English. Participants recruited through Prolific received a small incentive after completing the questionnaire and passing the plausibility check.

A total of 707 individuals completed all sections of the survey (384 German-speaking, 323 English-speaking). However, some were excluded for indicating they did not regularly spend time with children or completing the survey for a child outside the specified age range (*n* =  21), completing less than half of the CHIMUS survey (*n* =  2), failing the attention check (*n* =  140), or providing an unassignable participant code (*n* =  2).

The final German-speaking sample consisted of 296 participants (148 female, 148 male) with a mean age of 33.13 years (*SD* =  9.87). 133 of the participants (44.9%) reported residing in the same household as the child. The children for whom the survey was completed had a mean age of 6.25 years (*SD* =  2.15). Regarding professional background, 35 (11.8%) had completed a pedagogical degree, 41 (13.9%) had received pedagogical training and 100 (37.2%) regularly worked professionally with children. In terms of musical background, 9 (3.0%) participants had a degree in music and 26 (8.8%) had completed professional training in music. Gold-MSI musical training scale values ranged from 7 to 49 (*M* =  22.66, *SD* =  10.22), with 5.5% receiving the minimum score and reporting no musical training.

The final English-speaking sample consisted of 246 participants (160 female, 84 male, 2 diverse) with a mean age of 37.35 years (*SD* =  9.43). Nearly half of the participants (48%, or 118 individuals) reported residing in the same household as the child. The children for whom the survey was completed had a mean age of 6.52 years (*SD* =  2.29). Most participants were UK residents (63.4%), with the remainder from other predominantly Western, English-speaking countries (Canada: 13.8%, Australia: 8.5%, USA: 8.1%, Ireland: 3.3%, New Zealand: 2.8%). Regarding professional background, 62 (25.2%) had completed a pedagogical degree, 35 (14.2%) had received pedagogical training and 110 (44.7%) regularly worked professionally with children. In terms of musical background, 7 participants had a degree in music, while 11 had some professional training in music. Gold-MSI musical training scale values were between 7 and 44 (*M* =  20.51, *SD* =  10.63), with 12.2% receiving the minimum score and thus reporting having no musical training.

#### Materials.

We employed the 9-item CHIMUS in German or English, respectively, as developed in Study 1. With the aim of testing for convergent validity, we included two measures that indirectly or directly assess musicality in children: parents completed the preschool version of the Music@Home questionnaire, while educators and other individuals not living with the child completed the KOMPIK Musical Interests and Competencies scale.

We used the German [86] and English [11] versions of the Music@Home Preschool questionnaire, comprising 17 items encompassing Parental Beliefs, Child Engagement with Music, Breadth of Musical Exposure and Parent Initiation of Musical Behaviour. Both a general factor and subscale scores can be computed. The authors report a Cronbach´s alpha value of .82 for the total scale (subscales between .66 and .79) for the German and .85 (subscales between .66 and .80) for the English version (for more detailed information on psychometric properties, see [11,86]).

For educators and other individuals not residing with the child, we used the KOMPIK Musical Interests and Competencies scale, in its original German version [67], as well as an English version for English-speaking participants. The translation was conducted by the authors of this manuscript and verified by a native English speaker. The KOMPIK Musical Interests and Competencies scale is part of a comprehensive battery for the observation of children aged 3.5 to 6 years that can be used by educational professionals in childcare facilities. The full version of the KOMPIK comprises 158 observation questions assigned to 11 developmental and educational areas, all pertaining to children’s skills, interests, and well-being. One of the developmental domains covered by KOMPIK is music. Musical interests and competencies are further subdivided into two different subscales. The Musical Interests subscale gauges interest in or commitment to musical activities encompassing both music reception and active participation. Observation questions within the Musical Competencies subscale primarily pertain to the child’s active approach to music, including their ability to produce music and receptive skills such as the ability to distinguish between different volumes and pitches. The authors report an internal consistency of *α* = .92 for the combined scores of the two subscales, indicating a very high level of reliability in measuring the underlying construct [57].

In addition, participants completed a demographic questionnaire. To assess their musicality, we included the Gold-MSI Musical Training subscale in its German [90] or English version [9], respectively.

#### Procedure.

Participants recruited via Prolific first completed a brief screening questionnaire to assess their eligibility for the study. To qualify, participants needed to regularly spend time with at least one child aged between 3 and 10 years and be fluent in either English or German. Eligible participants were then invited to complete the survey, which included the Child Musicality Screening along with either the Music@Home questionnaire (for parents) or the KOMPIK items (for educators and other raters). Additionally, they responded to the Gold-MSI items, and provided demographic details. A random subsample was invited to retake the Child Musicality Screening two weeks later. This interval was chosen to balance minimising memory effects among raters while ensuring that any significant changes in the child’s musicality were unlikely. In total, 111 German-speaking participants (aged 18 to 70, *M* =  35.24, *SD* =  9.45; mean age of the children *M* =  6.37, *SD* =  2.08) and 89 English-speaking participants (aged 19 to 62, *M* =  37.16, *SD* =  8.80; mean age of the children *M* =  6.26, *SD* =  2.29) completed the survey on both occasions.

#### Statistical analyses.

Statistical analyses were performed using SPSS Statistics, version 29.0.1.0 [91] and RStudio, version 4.4.1 [70]. As in previous analyses, we recoded inverted items, calculated subscale values, and determined total scale values by summing the item values. All analyses were done separately for the German and English sample. We employed CFA to examine the factorial validity of the models. Internal reliability for each subscale, as well es for the overall Child Musicality Screening, was assessed using Cronbach’s alpha and MacDonald’s omega total. Pearson correlations were calculated for the test-retest reliability analysis. We also performed correlational analyses to evaluate the convergent validity of our instrument with the Music@Home and the KOMPIK Musical Interests and Competencies.

#### Results of study 3.

To assess the adequacy of the factor model, confirmatory fit indices were calculated and are presented in Table 9. The fit indices for the German data fall within the acceptable to good range, while the English data demonstrates a very good fit to the model.

**Table 9:** Confirmatory factor analysis for the English and German version of the Child Musicality Screening.

Models	χ²	df	RMSEA	CFI	TLI	SRMR
CHIMUS German	60.40	24	.072	.956	.934	.046
CHIMUS English	35.73	24	.045	.987	.981	.037

RMSEA =  Root Mean Square Error of Approximation, CFI =  Comparative Fit Index, TLI =  Tucker-Lewis Index, SRMR =  Standardized Root Mean Square Residual.

Table 10 presents the internal reliability estimates for the Child Musicality Screening in both its German and English versions, as indicated by Cronbach’s alpha and MacDonald’s omega total. Overall, the tool exhibits moderate to very good internal consistency, with consistently better results observed in the English version. The Production subscale in the German version showed the lowest reliability, while in the English version, although still acceptable, it was also lower than that of the other subscales. The test-retest reliability for the total scale was very high for both language versions (.88 for German and .86 for English, both with p-values < .001). Most subscales also showed very high test-retest correlations, though the Production subscale in the English version had a good, but somewhat lower, test-retest correlation (see Table 10 for details).

**Table 10:** Estimates of internal reliability (Cronbach’s alpha, MacDonald’s omega total) and test-retest correlations for the general factor and subscales of the German and English version of the Child Musicality Screening.

	α	ω_*tot*_	test-retest
CHIMUS German Total Scale	.80	.87	.88***
Enthusiasm & Motivation	.79	.80	.81***
Perception	.81	.81	.72***
Production	.65	.69	.76***
CHIMUS English Total Scale	.84	.90	.86 ***
Enthusiasm & Motivation	.82	.83	.81***
Perception	.85	.85	.85***
Production	.73	.75	.66***

α =  Cronbach’s Alpha [84], ω_*tot*_ =  Omega total [85], test-retest =  Pearson correlation (2-tailed), *** =  *p* < .001.

As shown in Table 11, convergent validity analysis revealed moderate to strong correlations between the CHIMUS and the general factor of the M@H questionnaire, as well as very strong correlations to the total scale of the KOMPIK. These results underscore the CHIMUS as a robust measure of musicality that aligns well with established instruments. Notably, as predicted, the correlations with the KOMPIK were generally stronger than those with the M@H, since the M@H scale primarily captures the musical home environment rather than focusing specifically on child musicality. Both the German and English versions of the CHIMUS questionnaire demonstrated significant correlations with various subscales of the M@H and the KOMPIK, further establishing its convergent validity. The Enthusiasm and Motivation subscale, in particular, showed strong correlations with the M@H Child Engagement with Music subscale and very strong correlations with the KOMPIK Musical Interest subscale, especially in the German version, where these relationships appeared more pronounced. The correlations align with expectations, with higher values observed for conceptually closer subscales (e.g., CHIMUS Enthusiasm & Motivation with M@H Child Engagement with Music) and lower values for more distinct subscales (e.g., CHIMUS Production with M@H Parental Beliefs). These findings suggest that the Child Musicality Screening effectively captures a broad range of musical behaviours and competencies in children, with the German version showing slightly stronger alignment with established instruments like the M@H and the KOMPIK.

**Table 11:** Convergent validity of the Child Musicality Screening with the Music@Home preschool and the KOMPIK.

	M@H General factor	M@H Parental beliefs	M@H Child engagement with music	M@H Parent initiation of musical behaviour	M@H breadth of musical exposure	KOMPIK Musical interest and competencies total scale	KOMPIK Musical interest	KOMPIK Musical competencies
CHIMUS German	.58*** (132)	.45*** (133)	.60*** (133)	.35*** (132)	.27** (133)	.73*** (148)	.73*** (153)	.57*** (157)
Enthusiasm & Motivation	.66*** (132)	.49*** (133)	.69*** (133)	.44*** (132)	.33*** (133)	.57*** (148)	.75*** (153)	.31*** (157)
Perception	.33*** (132)	.36*** (133)	.32*** (133)	.17 * (132)	.11 (133)	.60*** (148)	.52*** (153)	.54*** (157)
Production	.29** (132)	.26** (133)	.30*** (133)	.15 (132)	.13 (133)	.49*** (148)	.40*** (153)	.45*** (157)
CHIMUS English	.41*** (118)	.36*** (118)	.40*** (118)	.19 * (118)	.22 * (118)	.71*** (128)	.66*** (128)	.62*** (128)
Enthusiasm & Motivation	.49*** (118)	.35*** (118)	.50*** (118)	.36*** (118)	.21 * (118)	.64*** (128)	.72*** (128)	.47*** (128)
Perception	.26** (118)	.33*** (118)	.19 * (118)	.03 (118)	.18 (118)	.64*** (128)	.53*** (128)	.60*** (128)
Production	.18 (118)	.14 (118)	.23 * (118)	.05 (118)	.11 (118)	.39*** (128)	.30*** (128	.38*** (128)

Pearson correlations (two-tailed). Values in parentheses indicate sample size (*N*). CHIMUS =  Child Musicality Screening, M@H =  Music@Home, Parent init. of mus. behaviour =  Parent initiation of musical behaviour, CBQ =  Child Behaviour Questionnaire. *  =  *p* < .05; ** =  *p* < .01; *** =  *p* < .001. Sample sizes (*N*) vary due to missing data in some variables.

## Discussion

The aim of the present paper was to develop and evaluate a short screening questionnaire to assess individual profiles of general musicality in children aged 3 to 10 years. Building on previous research into conceptions of musicality [7,51], the instrument was designed to cover a wide range of musical behaviours and abilities, reflecting the multifaceted nature of musicality in children. In addition, we aimed to make the instrument available in English and German to increase its accessibility and facilitate comparability in future studies.

To this end, we conducted three studies. In Study 1, an initial pool of items was condensed on the basis of factor analysis and invariance tests for languages and age. The result of Study 1 was the 9-item version of the questionnaire, which we called the Child Musicality Screening. In Study 2, we obtained preliminary evidence of the reliability and validity of the screening in a separate German-speaking sample. To this end, we assessed a parent and a teacher rating for each child. In Study 3, we established retest reliability and conducted more detailed analyses of convergent validity for both the German and the English versions.

Our results indicate that, according to ratings by parents and educators, children’s musicality is best characterised by a three-factor model. The model showed scalar invariance for younger and older children and for both language versions. The factors were substantially correlated and corresponded to the dimensions Enthusiasm and Motivation for music, Music Perception, and Music Production.

### Enthusiasm and motivation

The factor Enthusiasm and Motivation captures a child’s intrinsic motivation and emotional engagement with music, their overall excitement and enjoyment in creating and incorporating music into their everyday activities. Although prior research highlights enthusiasm and motivation as crucial elements of both adult [5,44,51] and child musicality [7,8], these dimensions are rarely included in musicality assessments, hindering the understanding of how emotional engagement influences musical development. Motivation for music-making emerges from complex interactions between individuals and their environment [92]. While research on the heritability of musical sensibility suggest that genetic factors may lay a foundation for musical interest and commitment [93], early musical experiences [44,94,95], the quality of music education [96], and self-efficacy are also critical in shaping motivation and interest. These factors influence self-perception of musical competence and foster ongoing engagement with music [92]. Enjoyment and intrinsic motivation derived from positive musical experiences are essential for supporting sustained engagement and progress in both informal and formal music learning [92,97]. Incorporating these often-overlooked emotional and motivational dimensions into musicality assessments is essential for gaining a more comprehensive understanding of musical development.

The Enthusiasm and Motivation factor exhibited excellent internal consistency among educators and good reliability for parent ratings. It showed comparably high internal consistency in both the German and English versions of the scale. Additionally, the Enthusiasm and Motivation scale also had the highest interrater correlation between parents and educators (.43), making it the factor on which parents and educators were most in agreement. Test-retest reliability over a two-week interval was high, demonstrating that this factor can be measured reliably. Assessing Enthusiasm and Motivation through this subscale could provide valuable insights into its role in musical development and help to determine whether the criticism that motivation is short-lived [98] holds true.

In terms of convergent validity, the correlation between school grades in music and the Enthusiasm and Motivation factor was somewhat less pronounced, possibly indicating that grades may reflect learning outcomes rather than a child’s motivation to engage in music. As expected, the Enthusiasm and Motivation scale showed the strongest correlations with measures of children’s engagement and interest in music. It was highly correlated with the KOMPIK Musical Interest scale, but only moderately to the KOMPIK Musical Competencies scale. Similarly, the factor was highly correlated with the Child Engagement scale of the Music@Home questionnaire and moderately correlated with parent-centred scales such as Parental Beliefs and Parent Initiation of Musical Behaviour. These findings indicate that parental beliefs and behaviours are related to child enthusiasm and motivation. Previous research highlighted the importance of parental involvement in musical development, such as providing musical instruments, engaging with the child musically, or enrolling the child in music education programmes, although many parents underestimate the importance of their own musical interactions at home [99]. For instance, active parental involvement during practice sessions has been shown to correlate with children’s enjoyment of music and their musical progress [100]. Furthermore, parental influence extends beyond practical involvement to include the values, attitudes, and expectations that parents communicate regarding their children’s musical growth [101–103].

Overall, these findings underline the importance of musical enthusiasm and motivation in children’s musical development and show that this factor can be reliably measured by our screening questionnaire. Despite its importance, this facet of musicality is often neglected in the assessment of musical ability. However, some researchers have emphasised its importance in identifying musical talent [95] and in achieving high levels of expertise [92,97]. The Enthusiasm and Motivation subscale provides a valuable measure of this essential dimension, allowing for a more comprehensive understanding of children’s musicality that goes beyond traditional assessments that focus on perception and production. By considering enthusiasm and motivation as integral components of a child’s musicality, new pedagogical approaches could be developed to increase engagement and foster overall musical growth. In research, assessing musical motivation opens up avenues for understanding its impact on long-term musical development.

### Music perception

The Music Perception factor reflects traditional views of children’s musicality as having a sense of timing and rhythm, a feeling for the beat, and a musical ear. Music perception has long been considered the most prominent candidate for assessing musical talent in children. In fact, since early in the last century, researchers have developed tests that assess music perception skills as indicators of musical ability. Prominent examples of test batteries with an emphasis on musical listening and perception skills include the Seashore Measures of Musical Talent [42], the Measures of Musical Abilities developed by Bentley [43], or the “Gordon tests” [104–108]. More recently developed test batteries include the Profile of Music Perception Skills [PROMS; 109], the Musical Ear Test [110], the Montreal Battery of Evaluation of Musical Abilities [MBEMA; 111], and individual tests that target specific musical skills [e.g., 112–114].

The Music Perception factor not only aligns with the emphasis on music perception in research, but also reflects musical developmental trajectories showing early and continuous growth. From birth, infants demonstrate significant perceptual abilities, related to rhythm [18–20] and pitch perception [21]. As children grow, their perceptual skills become more sophisticated, influenced by cultural exposure and listening experiences [35,115]. Some more complex abilities, like harmony [e.g., 22,116], tempo perception [117], and the recognition of more subtle pitch and sound characteristics develop gradually, reaching reach adult-like proficiency in late childhood or adolescence [23,33]. This underscores the importance of including the perception factor in the Child Musicality Screening to enhance our understanding of music perception development.

The Music Perception factor demonstrated excellent internal consistency in the educator sample and good reliability in the parent ratings (Study 2). In Study 3, this factor also showed good internal consistency across both the German and English samples, reinforcing these findings. The agreement between parents and educators was moderate, which is acceptable given the abstract content of the items (e.g., “the child has a feeling for the beat”), and in comparison to similar studies with low interrater reliabilities between parents and teachers [118–121]. Test-retest reliability was very high, indicating the robustness of the scale. Convergent validity with school grades in music was moderate, suggesting that while the Music Perception factor is related to students’ academic performance in music, the correlation is not very pronounced. The Music Perception factor was also only moderately related to the Music@Home scale (weakly related in the English sample), indicating a limited relationship with the musical home environment. However, it showed stronger correlations with the KOMPIK scales, which assess musical interest and competencies and are conceptually closer to the CHIMUS than the Music@Home scale.

Overall, the Music Perception factor effectively captures traditional and essential dimensions of child musicality with reliable measurement properties. Its inclusion in the Child Musicality Screening is essential for assessing musical profiles in children, as music perception undergoes significant development from early to late childhood and serves as a critical foundation for the acquisition of production skills and more complex musical abilities. As such, it is key to understanding and supporting children’s musical growth. While further research is needed to explore the convergent validity of the scale with behavioural tests of music perception, the Child Musicality Screening offers a highly time-efficient method for assessing this aspect of musicality. These findings underscore the factor’s value in providing a robust assessment of a core element of musicality, which is foundational to understanding and fostering children’s musical development.

### Music production

The Music Production subscale addresses the challenges children face in producing or reproducing music, such as singing in tune, maintaining a beat, or playing an instrument. Although music production has been shown to be an important component of conceptions of musicality [7,8,51], tests of music production are less common than tests of musical perception. Recent research emphasises the need to include music production in comprehensive musicality assessments [6,7,122,123]. Music production is crucial to assess because its development builds on perceptual skills and progresses throughout childhood [23]. Moreover, music production is evident from very early in development [24,25,29] and children are actively and naturally engaged in musical practices long before formal instruction begins [34,124]. However, certain abilities in singing and rhythm production, only fully develop in late childhood, with some requiring formal training [32]. Thus, music production provides valuable developmental insights, making it an essential component of the Child Musicality Screening. Hence, music production’s developmental trajectory offers critical variation to capture, explore, and explain, making it an essential component of the Child Musicality Screening.

The factor Music Production exhibited slightly lower psychometric benchmark values compared to the other two scales, particularly in terms of internal consistency. Specifically, the internal consistency was high for educators and acceptable for parents, whereas the other scales showed good to excellent reliability across both groups of participants. In Study 3, the internal consistency of the German version of the Production scale was just below .70, reflecting marginally acceptable values, while the English version had acceptable reliability (>.70). Interrater correlation was moderate and comparable to the perception scale. Test-retest reliability was very strong for the German sample but moderate for the English sample. Convergent validity with school grades was established, with the Production scale showing the highest correlation (though still moderate at .40) with music grades compared to the other subscales. This suggests that the ability to produce or reproduce music is more closely related to school grades in music than the other two subscales. The correlation with the Music@Home scale was low for the German version and not significant for the English version, indicating that the Production scale measures a distinct aspect of musical ability. Convergent validity with the KOMPIK scale was moderate for both versions, with slightly higher values for the German version.

The slightly lower or inconsistent values in psychometric criteria could be due to several factors. Notably, the subscale consists exclusively of negatively worded items. This unintentional clustering during factor analysis may have introduced method effects, potentially leading to respondent confusion and impacting the consistency of their responses. Furthermore, the inherent complexity of evaluating music production, particularly in children, could contribute to these issues. Unlike more universally understood constructs like enthusiasm and motivation, music production involves nuanced judgments of various musical features such as key, timing, rhythm, intonation, and creative expression. This complexity might result in increased variability in ratings, particularly when evaluators lack musical training. Existing literature supports the notion that musical performance evaluation is often affected by interrater variability due to its subjective aspects, even in trained observers [e.g., 125,126]. However, unlike positive attributes, which can be abstract and challenging to evaluate, deficits in music production might be more apparent and easier to detect. Rating music production abilities based on difficulties may therefore provide a more accurate and practical method for evaluating skills in creating and reproducing music.

Overall, the Music Production subscale offers valuable insight into a key aspect of children’s musicality. Its inclusion in the Child Musicality Screening is essential for understanding the role of music production in musical development and its links to motivation and music perception. The screening captures the critical developmental period when many of the children’s production abilities reach levels comparable to those of untrained adults and when some children begin instrumental lessons, marking the start of more structured music production and practice. The subscale showed slightly lower psychometric values than the other factors, possibly due to the negative wording of the items and the inherent complexity of assessing music production. The use of positively formulated items may be a better solution, as it could enhance the clarity and reliability of the responses. Testing this in a follow-up study would be valuable to determine if such changes improve the subscale’s psychometric properties. In order to validate the subscale more robustly, future studies should also examine its correlations with behavioural music production tests.

### Validity

The development of the Child Musicality Screening involved rigorous testing to ensure both its validity and reliability. Content validity was established in Study 1 through a comprehensive item generation process. A substantial proportion of the items were derived from Buren et al. [7], whose item pool was informed by free-text responses from a diverse group of individuals describing their understanding of musical ability [5], and a subsequent study [51]. This approach reflects musicality as a socially constructed concept. To ensure a comprehensive item pool, additional items were drawn from the literature on musical development. This thorough process ensured that the initial item pool covered key dimensions of child musicality, including rhythmic ability, pitch recognition, musical communication, and musical engagement.

Construct validity was evaluated through exploratory and confirmatory factor analyses conducted in Studies 1–3. The final three-factor structure—comprising Enthusiasm and Motivation for music, Music Perception, and Music Production—aligned well with existing models, particularly those focused on conceptions of child musicality [7,8]. These studies suggest that while musicality in childhood is multifaceted, it consists of distinct components that differ from those in adults. Interestingly, while previous research on adult musicality [51] identified a greater number of components, our results confirm that a more streamlined structure is sufficient for capturing the essence of children’s musical development without oversimplifying it. In line with the identification of Enthusiasm and Motivation as a critical component of children’s musicality [7,8], our findings emphasise enthusiasm and motivation as a central element of children’s musicality, as evidenced by this being one of three core factors. The separation of Music Perception and Music Production as distinct but interrelated factors further aligns with previous studies on child musicality, which also recognise these as essential components of musical abilities [though combined into a single composite component in previous work; 7,8]. However, the components of Musical Communication and Analytical Understanding that were important components in two previous surveys with stakeholders [parents, educators, music teachers; 7,8] did not emerge as distinct factors in our final screening questionnaire. This may be due to the abstract nature of these components, which makes them difficult for non-experts to assess within a short screening tool. In addition, the previous study asked participants about a hypothetical musical child, whereas our study focuses on typical children, suggesting that these qualities may only become more relevant or observable at higher levels of musicality.

Invariance tests confirmed scalar invariance across different age groups and across the German and English versions of the questionnaire, indicating that factor loadings and item intercepts were consistent across age and language groups. This underlines the robustness of the construct and its cross-cultural applicability in modern Western contexts.

Criterion validity was assessed in Studies 2 and 3 by examining correlations between the CHIMUS and other measures of musicality. In Study 2, correlations with the reported school grade showed a moderate correlation with the CHIMUS Total scale. The German version of the CHIMUS showed a strong correlation with the Music@Home scale in Study 2, which was replicated in Study 3 with an independent sample of participants. The English version of the CHIMUS showed a more moderate correlation with the Music@Home General factor. Very strong correlations were also observed between the CHIMUS and KOMPIK total scales for the German and English versions. At the subscale level, conceptually more similar subscales (e.g., CHIMUS Enthusiasm and Motivation and M@H Child Engagement with Music) showed stronger associations than more divergent subscales. These strong associations, especially when replicated with an independent sample, provide robust evidence for the convergent validity of the CHIMUS. Furthermore, divergent validity was found in Study 2, where CHIMUS scores were not significantly related to Extraversion, as assessed by two different personality/temperament inventories (BFI-10 and CBQ).

### Reliability

Regarding reliability, the CHIMUS demonstrated generally high internal consistency across all three studies. Cronbach’s alpha and Omega total values for the overall scale and each subscale mostly exceeded the commonly accepted threshold of .70, indicating that the items within each factor reliably measure the same underlying construct. Separate analyses of the internal consistency were conducted for parents and educator ratings (Study 2) and for the German and English version (Study 3). In Study 2, the comparison between educators and parents revealed good to excellent reliability for the educator sample, while the internal consistency for the parent sample was in an acceptable to good range. In Study 3, the internal consistency was also generally high, with the exception of the Production subscale, which was slightly below .70 for the German version and acceptable for the English version. Analyses of internal consistency for the different groups (parents vs. educators) and versions (German vs. English) thus revealed good to excellent reliability in most cases, with educator ratings being particularly consistent, and the English version showing slightly better reliability than the German version in Study 3.

To explore interrater reliability, differences between teacher and educator ratings were analysed to assess consistency across raters. Interrater reliability showed moderate correlations, with the Motivation subscale exhibiting high interrater reliability. While some discrepancies between ratings were anticipated due to potential parental bias—where parents may rate their children more favourably—the correlations were strong enough to confirm the CHIMUS’s reliability across different rater groups. This is notable given the typically low interrater reliability found in similar studies [118–121].

Interestingly, the model exhibited better fit parameters when ratings were provided by professionals (teachers/educators) compared to parents. This suggests that the CHIMUS may yield more reliable results when used by individuals with pedagogical expertise, probably due to the more objective perspective of educators and their ability to compare a wider range of children. Despite the careful design, including the use of negatively worded items, there remains the possibility of response bias such as acquiescence or social desirability, particularly in parent-completed questionnaires. Parents may tend to rate their children more positively, particularly in terms of ability, which could affect the accuracy of the ratings and the overall validity of the results. However, this positive bias may be less pronounced in the motivation domain than in other subscales, which may explain the slightly higher interrater reliability compared to the other two subscales. Admitting a lack of motivation in one’s own child may be easier for parents than admitting deficits in ability. In addition, motivation may be more readily assessed by non-experts, making it somewhat easier for laypersons to evaluate compared to other aspects of musicality.

Test-retest reliability was assessed in Study 3 over a two-week interval, showing strong stability of the questionnaire scores (*r* = .85). This suggests that the CHIMUS produces consistent results over time, which is crucial for longitudinal studies and repeated assessments.

### Limitations and future studies

Although the Child Musicality Screening has demonstrated strong validity and reliability, further research is essential to extend its utility and address some of its limitations. Although the CHIMUS showed good convergent validity with other questionnaires, future studies should aim to increase its external validity by comparing it with standardised tests of music perception and production. In addition, a more detailed analysis of how different raters—such as parents, teachers, and music educators—assess musicality could provide valuable insights. Understanding the variability in ratings between these groups will help to identify potential biases and ensure that the CHIMUS remains effective in different assessment contexts.

Given that the current study primarily involved participants from Western cultural backgrounds, the generalisability of the findings may be limited. The CHIMUS may not fully capture the nuances of musicality in children from non-Western or underrepresented communities. To address this, future research should focus on cross-cultural validation. This would involve adapting the questionnaire for use in non-Western and culturally diverse settings and then assessing its validity and reliability in these contexts. Such efforts would determine whether the construct of musicality is consistent across different cultural backgrounds and highlight any necessary adjustments to the instrument.

The CHIMUS has been validated across two age groups (3 to 6 years and 7 to 10 years), but its applicability to younger children (under 3 years) or older adolescents remains unexplored. Future studies could investigate whether the CHIMUS, either in its original or a modified form, can effectively assess musicality in toddlers and adolescents. In addition, longitudinal research would be invaluable in understanding how musicality develops over time. By following musical development from early childhood to adolescence, researchers can examine how early musical characteristics influence long-term skills and related outcomes, such as academic achievement and cognitive development.

The CHIMUS provides a broad overview of musicality and its development, offering valuable insights into general aspects of musical ability and its constituent facets. In order to gain a more detailed understanding of specific developmental changes, additional tests or questionnaires may be necessary. Future studies could investigate how supplementary measures could complement the CHIMUS, with the potential to yield a more comprehensive understanding of musical development across various dimensions.

In summary, the Child Musicality Screening provides researchers and educators with a novel, efficient and reliable tool for the systematic assessment of children’s musicality, filling a critical gap in the tools available to assess children’s musical development. It is based on current conceptualisations of child musicality, has good psychometric properties and can be applied across different stages of childhood, allowing researchers to explore a wide range of questions related to child development. In addition, the screening facilitates the identification of patterns and trajectories of musical growth, supporting more nuanced investigations of the factors that influence musical ability. By aligning with current conceptualisations of musicality and adhering to rigorous psychometric standards, this tool contributes to a more unified framework for assessing musical ability in children. This data-driven approach not only advances research, but also provides practical benefits for educators. The ability to assess individual musical profiles allows for more tailored educational approaches, enabling educators to better support each child’s musical development. By understanding how children’s musical skills develop over time, educators can tailor their music education support to better meet the need of individual students, leading to more effective teaching strategies and interventions. Additionally, identifying children with exceptional musical abilities can facilitate targeted interventions and enrichment opportunities to further develop their musical aptitude.

## Supporting information

S1 File Initial item list. The initial item list of the questionnaire in both German and English, with original sources.
(PDF)

S2 File Child Musicality Screening (English version).
The final English version of the Child Musicality Screening.
(PDF)

S3 File Child Musicality Screening (German version).
The final German version of the Child Musicality Screening.
(PDF)
