# Supplementary material for: Screening musicality in children: Development and initial validation of a new tool for rapid assessment of musical profiles
Source: PLoS One. 2025 Mar 5;20(3):e0317962. doi: 10.1371/journal.pone.0317962 (PMC11882079; doi:10.1371/journal.pone.0317962)
Supplement: S1 File — The initial item list of the questionnaire in both German and English, with original sources. (PDF) [file pone.0317962.s001.pdf]

Initial Item List of the Child Musicality Screening

	German Items	English Items	Original Source
**1**	…hat ein gutes Gespür für Timing und Rhythmus.	…has a good sense of timing and rhythm.	Buren et al., 2021
**2**	…besteht nicht auf seinem eigenen Willen beim Musizieren und Genießen von Musik.	…does not insist on his/her own will when making and enjoying music.	Buren et al., 2021
**3**	…genießt es, zu singen, und macht dies oft.	…enjoys singing and does so frequent.	Buren et al., 2021
**4**	…zeigt kein Interesse daran, Töne in verschiedenen Umgebungen zu erkunden.	…does not seem curious to explore sounds in different environments.	Burke, 2018
**5**	…ist in der Lage, Unterschiede zwischen verschiedenen Arten von Musik zu hören und zu untersuchen.	…is able to hear, compare and distinguish different types of music.	Buren et al., 2021
**6**	…kann genau zuhören und passend musikalisch reagieren.	…can listen carefully and react appropriately to music.	Buren et al., 2021
**7**	…ist in der Lage, Klang, Tonhöhe und Rhythmen zu verinnerlichen.	…is able to internalize sound, pitch and rhythm.	Buren et al., 2021
**8**	…benutzt keine neutralen Objekte , um damit Musik zu machen.	…doesn´t repurpose music-neutral objects into musical instruments.	Buren et al., 2021 open answer
**9**	…lässt sich nicht vollkommen auf Klänge ein.	…won´t let him-/herself become immersed in sounds.	Buren et al., 2021
**10**	…ist in der Lage, im Takt mit anderen zu musizieren, indem es Zuhören und Klangerzeugung kombiniert.	…is able to play music in time with others by listening and sound production.	Burke, 2018
**11**	…ist sehr musikalisch.	…is very musical.	Buren et al., 2021
**12**	…kann beim Singen oder einfachen Musizieren Emotionen ausdrücken, die ein Zuhörer versteht.	…can communicate emotions to a listener when singing and making music.	Buren et al., 2021
**13**	…hat eine große Affinität zur Musik.	…has a great affinity for music.	Buren et al., 2021
**14**	…kann Melodien oder Rhythmen erfinden, entweder mit der Stimme, oder einem einfachen Instrument.	…can invent melodies or rhythms, either with his/her voice or a simple instrument.	Buren et al., 2021
**15**	…stellt keine Verbindung zwischen Musik und Charakteren und Geschichten her.	…does not associate music with characters and stories.	Burke, 2018
**16**	…hat eine gute Hörfähigkeit, z.B. für Melodien und Rhythmen.	…has good hearing ability, e.g. for melodies and rhythms.	Buren et al., 2021
**17**	…scheint in Musik, die es hört, die Stimmung oder darin transportierte Gefühle nicht wahrzunehmen.	…does not seem to pick up on emotions conveyed by music.	Buren et al., 2021
**18**	…hat ein Gefühl für den Takt.	…has a feeling for the beat.	Buren et al., 2021
**19**	…versucht nicht, sich musikbezogene Objekte wie Instrumente oder Abspielgeräte anzueignen.	…does not seek to aquire music related objects.	Krause, 2017
**20**	…kann Melodien gut reproduzieren.	…can reproduce melodies well.	Buren et al., 2021
**21**	…bewegt sich oft nicht synchron zur Musik.	…often moves out of sync with music.	Buren et al., 2021
**22**	…kann auf die Stimmung einer Melodie reagieren.	…is able to react to the mood of a melody.	Buren et al., 2021
**23**	…ist sich verschiedener Aspekte von Musik bewusst, so dass man mit ihm über die Instrumente eines Stückes oder die ausgelösten Gefühle sprechen kann.	…is aware of different aspects of music, so you can talk to him/her about the instruments in a piece or the emotions evoked by it.	Heller & Perleth, 2007
**24**	…ist motiviert, Musik zu machen, sodass es dabei immer versierter wird.	…is motivated to make music, so that he/she becomes more and more skilled.	Buren et al., 2021
**25**	…empfindet keine Freude daran, Zeit in musikalischen Umgebungen zu verbringen.	…does not enjoy spending time in musical environments.	Buren et al., 2021
**26**	…schätzt verschiedene Arten von Musik.	…appreciates different kinds of music.	Buren et al., 2021
**27**	…kann der Struktur der Musik, die es hört, folgen und darüber sprechen.	…can follow the structure of the music he/she listens to, and talk about it.	Buren et al., 2021
**28**	…hat keine Assoziationen zwischen Musik und Routinen (hat beispielsweise kein "Händewaschlied").	…has no association between music and routines (e.g. does not have a song for washing his/her hands).	Burke, 2018
**29**	…zeigt kaum Interesse daran, sich an Muster zu erinnern.	…shows little interest in memorising patterns.	Buren et al., 2021
**30**	…macht keine Musiker:innen oder Sänger:innen nach und imitiert diese nicht.	…does not mimic or imitate musicians or singers.	Krause, 2017
**31**	…hat oft den Wunsch, Musik zu machen.	…often has the desire to make music.	Buren et al., 2021
**32**	…kann sich durch Klang selbst ausdrücken.	…can express him-/herself through sound.	Buren et al., 2021
**33**	…hat Schwierigkeiten, Musik zu produzieren oder zu reproduzieren.	…shows difficulties in producing or reproducing music.	Buren et al., 2021
**34**	…erfreut sich an der Beschäftigung mit Musik.	…enjoys being occupied in musical activities.	Buren et al., 2021
**35**	…hat Probleme, Melodien, die es zuvor gehört hat, zu reproduzieren.	…has issues reproducing melodies he/she has heard before.	Heller und Perleth, 2007
**36**	…hört gerne Musik und kann sie mit Worten und Gesten beschreiben.	…likes listening to music and can describe it with words and gestures.	Buren et al., 2021
**37**	…zeigt Schwierigkeiten darin, mit einem offenen Geist auf Musik zuzugehen.	…displays challenges in experiencing music with an open mind.	Buren et al., 2021
**38**	…erkennt keine Komponist:innen oder Sänger:innen wieder.	…does not recognise composers or singers.	Buren et al., 2021 open answer
**39**	…findet rhythmische Muster in seiner Umgebung.	…finds rythmical patterns in his/her enviroment.	Buren et al., 2021 open answer
**40**	…passt beim Musikmachen wenig auf, sodass es nicht merkt, ob es so klingt wie beabsichtigt.	…pays little attention while making music, so he/she does not realize if it sounds as intended.	Buren et al., 2021
**41**	…kontrolliert die Auswahl der Musik, die es hört, indem es angibt, welche Musikaufnahmen es hören möchte.	…controls his/her listening choices by indicating which recorded music he/she wants to listen to.	Burke, 2018
**42**	…würde sich eher mit Musik ausdrücken als mit anderen Mitteln.	…would rather express him-/herself through music than through other means.	Buren et al., 2021
**43**	…kann mit anderen durch Musik kommunizieren, indem es musikalische Geräusche macht, zuhört, improvisiert, tanzt und Musik versteht.	…can communicate with others by producing musical sounds, listening, improvising and dancing.	Buren et al., 2021
**44**	…interessiert sich für Musik.	…is interested in music.	Buren et al., 2021
**45**	…bringt mehrere Fähigkeiten zusammen: Es hat musikalische Ideen und versucht, sie umzusetzen.	…brings several skills together: Has musical ideas and tries to implement them.	Buren et al., 2021
**46**	…unterscheidet nicht zwischen verschiedenen Musikinstrumenten.	…does not distinguish between different musical instruments.	Krause, 2017
**47**	…zeigt wenig Motivation, mit anderen zu musizieren.	…demonstrates little motivation to make music with others.	Krause, 2017
**48**	…hat Interesse daran, Musikinstrumente zu spielen.	…shows interest in playing a musical instrument.	Buren et al., 2021 open answer
**49**	…genießt Musikmachen als Teil seines Lebens.	…enjoys making music a part of his/her life.	Buren et al., 2021
**50**	…hat große Begeisterung für Musik.	…has great enthusiasm for music.	Buren et al., 2021
**51**	…ist beim Musikmachen kreativ.	…is creative when making music.	Buren et al., 2021
**52**	…hat Schwierigkeiten damit, Melodien und Tonverläufe zu erkennen.	…has difficulties in recognizing melodies and tone progression.	Krause, 2017
**53**	…macht gerne spontan Musik oder musikalische Geräusche.	…likes to spontaneously produce music or musical sounds.	Buren et al., 2021
**54**	…lässt sich leicht ablenken, wenn es mit musikalischen Aktivitäten beschäftigt ist.	…is easily distracted when engaged with musical activities.	Krause, 2017
**55**	…wendet Kriterien an, um Musik zu bewerten.	…applies criteria to evaluate music.	Buren et al., 2021
**56**	…erkennt keine Melodien wieder.	…does not recognise melodies.	Heller & Perleth, 2007
**57**	…zeigt beim Tanzen zur Musik oder beim eigenen Musizieren, dass es Muster erfassen kann.	…can pick up patterns when dancing or making music.	Buren et al., 2021

References:

Buren V, Müllensiefen D, Roeske TC, Degé F. What makes a child musical? Conceptions of musical ability in childhood. Early Child Dev Care. 2021;191(12):1985-2000.

Burke N. Musical Development Matters in the Early Years. Brit Assoc Early Child Educ. 2018.

Heller KA, Perleth C. MHBT-P - Münchner Hochbegabungstestbatterie für die Primarstufe (Münchner Hochbegabungs-Testbatterie für die Primarstufe). 2007.

Krause M. Kompetenzen und Interessen von Kindern (KOMPIK). In: Erpenbeck J, von Rosenstiel L, Grote S, Sauter W, editors. Handbuch Kompetenzmessung. 3rd ed. Stuttgart: Schäffer-Poeschel; 2017. pp. 355-61.
