# Supplementary material for: Screening musicality in children: Development and initial validation of a new tool for rapid assessment of musical profiles
Source: PLoS One. 2025 Mar 5;20(3):e0317962. doi: 10.1371/journal.pone.0317962 (PMC11882079; doi:10.1371/journal.pone.0317962)
Supplement: S2 File — The final English version of the Child Musicality Screening. (PDF) [file pone.0317962.s002.pdf]

MAX PLANCK INSTITUTE
FOR EMPIRICAL AESTHETICS

Please think about the child for each of the following questions and answer each statement on a scale from 1 ("totally disagree" or "never") to 5 ("totally agree" or "always"). There are no right or wrong answers, please select the answer that best applies to the child.

The child…

	**1 totally disagree**	**2 partly disagree**	**3 neither agree nor disagree**	**4 partly agree**	**5 totally agree**
...has a good sense of timing and rhythm.	☐	☐	☐	☐	☐
...often has the desire to make music.	☐	☐	☐	☐	☐
...has a feeling for the beat.	☐	☐	☐	☐	☐
...has good hearing ability, e.g. for melodies and rhythms.	☐	☐	☐	☐	☐

The child…

	**1 never**	**2 rarely**	**3 sometimes**	**4 often**	**5 always**
...shows difficulties in producing or reproducing music.	☐	☐	☐	☐	☐
...has great enthusiasm for music.	☐	☐	☐	☐	☐
...pays little attention while making music, so he/she does not realise if it sounds as intended.	☐	☐	☐	☐	☐
...enjoys making music as part of his/her life.	☐	☐	☐	☐	☐
...has issues reproducing melodies he/she has heard before.	☐	☐	☐	☐	☐

Thank you very much for your participation!
