# Supplementary material for: Screening musicality in children: Development and initial validation of a new tool for rapid assessment of musical profiles
Source: PLoS One. 2025 Mar 5;20(3):e0317962. doi: 10.1371/journal.pone.0317962 (PMC11882079; doi:10.1371/journal.pone.0317962)
Supplement: S3 File — The final German version of the Child Musicality Screening. (PDF) [file pone.0317962.s003.pdf]

Bitte denken Sie bei jeder der folgenden Fragen an das Kind und beantworten Sie jede Aussage auf einer Skala von 1 („stimme überhaupt nicht zu“ bzw. „nie“) bis 5 („stimme voll und ganz zu“ bzw. „immer“). Es gibt keine richtigen oder falschen Antworten, bitte wählen Sie die Antwort aus, die am ehesten auf das Kind zutrifft.

Das Kind…

	**1 Stimme überhaupt nicht zu**	**2 Stimme nicht zu**	**3 Weder/ noch**	**4 Stimme zu**	**5 Stimme voll und ganz zu**
...hat ein gutes Gespür für Timing und Rhythmus.	☐	☐	☐	☐	☐
...hat oft den Wunsch, Musik zu machen.	☐	☐	☐	☐	☐
...hat ein Gefühl für den Takt.	☐	☐	☐	☐	☐
...hat eine gute Hörfähigkeit, z.B. für Melodien und Rhythmen.	☐	☐	☐	☐	☐

Das Kind…

	**1 Nie**	**2 Selten**	**3 Manch-mal**	**4 Oft**	**5 Immer**
...hat Schwierigkeiten, Musik zu produzieren oder zu reproduzieren.	☐	☐	☐	☐	☐
...hat große Begeisterung für Musik.	☐	☐	☐	☐	☐
...passt beim Musikmachen wenig auf, sodass es nicht merkt, ob es so klingt wie beabsichtigt.	☐	☐	☐	☐	☐
...genießt Musikmachen als Teil seines Lebens.	☐	☐	☐	☐	☐
...hat Probleme, Melodien, die es zuvor gehört hat, zu reproduzieren.	☐	☐	☐	☐	☐

Herzlichen Dank für Ihre Teilnahme!
